# Chromatic‐Zone Mapping for Rapid Discovery of Antibacterial Alloys with Nanostructured Surfaces

**DOI:** 10.1002/advs.202513454

**Published:** 2025-10-24

**Authors:** Qiu‐Yu Zhao, Yu‐Ying Liu, Li‐Wei Hu, Hong‐Xi Duan, Ming‐Xing Li, Yan‐Hui Liu, Jing Jiang, Zhen Lu, Wei‐Hua Wang

**Affiliations:** ^1^ Institute of Physics Chinese Academy of Sciences Beijing 100190 China; ^2^ School of Health Sciences & Biomedical Engineering Hebei University of Technology Tianjin 300130 China; ^3^ College of Materials Science and Optoelectronic Technology University of Chinese Academy of Sciences Beijing 100049 China; ^4^ School of Physical Science University of Chinese Academy of Sciences Beijing 100049 China; ^5^ Songshan Lake Materials Laboratory Dongguan Guangdong 523808 China

**Keywords:** antibacterial performance, chromatic‐zone screening approach, high‐throughput dealloying, metallic glass

## Abstract

Addressing antibiotic‐resistant bacteria requires the efficient development of antibiotic‐free antimicrobial materials. Herein, a high‐throughput parallel chromatic‐zone screening strategy is developed that enables the simultaneous screening of composition and surface structure, thereby optimizing antimicrobial performance. As an example, a MgCuPdGd alloy library with diverse compositions and nanostructures, consisting of 229 samples with continuous compositional gradients and varied nanostructured morphologies is constructed by integrating magnetron co‐sputtering and chemical dealloying. The dealloyed samples exhibit distinct chromatic zones—red, yellow, and green—each associated with unique compositional and microstructural features. Among these regions, the red Cu‐rich region demonstrates the most outstanding antibacterial performance, achieving a 95% reduction of viable *Staphylococcus aureus* (*S. aureus*). Comprehensive characterization confirms that the superior antimicrobial efficiency originates from the synergistic contribution of the CuPd alloy and the optimized nanostructure. Furthermore, the observed correlation among surface color, composition, morphology, and antibacterial performance highlights the predictive capability of the chromatic‐zone screening approach, thereby reducing characterization requirements by 90% and enabling morphology and performance estimation across large material libraries. This work not only offers a rapid and cost‐effective strategy for identifying antimicrobial materials but also provides a versatile platform adaptable to the development of functional materials for broader biomedical and environmental applications.

## Graphical abstract

A high‐throughput parallel chromatic‐zone screening strategy that enables the simultaneous screening of alloy composition and surface structure. Ultimately linking surface color, composition, and morphology with antimicrobial performance enables the screening and prediction of color zones, thereby reducing characterization requirements by 90% and achieving predictive assessment of morphology and performance.

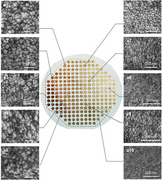

## 1 Introduction

Pathogenic bacteria pose a major global threat to public health, largely due to their ability to cause severe and life‐threatening infections.^[^ ^1^, ^2^, ^3^, ^4^, ^5^ ^]^ Although antibiotics remain one of the most effective treatments for bacterial infections, their widespread misuse has accelerated the emergence of antibiotic‐resistant strains.^[^ ^6^ ^]^ This crisis is further exacerbated by the sluggish development of new antibiotics and environmental contamination resulting from antibiotic overuse, creating an urgent need for alternative antimicrobial strategies.^[^ ^7^, ^8^, ^9^, ^10^, ^11^ ^]^ Inorganic antimicrobial agents offer promising solutions due to their high chemical stability, sustained bactericidal activity, and minimal risk of inducing resistance.^[^ ^12^, ^13^ ^]^ Among them, metallic nanomaterials, with unique physical and chemical properties, have emerged as exceptional antibacterial candidates, owing to their high surface area‐to‐size ratio and remarkable capability to penetrate microbial biofilm,^[^ ^14^, ^15^, ^16^, ^17^, ^18^ ^]^ enable multiple mechanisms of synergistic antimicrobial activity such as the release of ions, the generation of reactive oxygen species (ROS) and physical destructio.^[^ ^19^, ^20^, ^21^ ^]^

The antibacterial efficacy of inorganic materials is primarily governed by two key factors: chemical composition and microstructural features.^[^ ^22^, ^23^ ^]^ To fully exploit the potential of metallic nanomaterials, strategies that couple controllable nanostructuring with quantitative activity screening are required.^[^ ^24^, ^25^ ^]^ Compared with multi‐component crystalline alloys, which are prone to forming inhomogeneous nanostructures due to segregation during the dealloying process, amorphous alloys can flexibly regulate composition and possess a disordered atomic arrangement, enabling the formation of a homogeneous nanostructure and ultimately achieving synergistic antimicrobial effects of multi‐component alloys.^[^ ^26^, ^27^, ^28^, ^29^ ^]^ These advantages enlarge the design space but also render exhaustive trial‐and‐error impractical; accordingly, high‐throughput experimentation becomes essential to rapidly map composition‐structure‐activity relationships and to build reliable datasets for materials discovery. At the nanoscale, composition and morphology modulate light‐matter interactions to generate structural colors.^[^ ^30^, ^31^ ^]^ Harnessing this optical response as a surrogate readout converts color into an immediate, non‐destructive, and efficient screening metric, enabling rapid preliminary assessment of nanostructure or composition and providing a new pathway for predicting antibacterial materials.

In this work, we develop a parallel chromatic‐zone screening method that simultaneously considers both composition and microstructure, enabling the rapid characterization of 229 samples in a single test. The MgCuPdGd amorphous alloy system was selected as a model due to its typical glass‐forming ability (GFA) and compositional flexibility, which allow tailoring of the composition and formation of homogeneous architectures. In addition, Cu provides well‐known antimicrobial activity, while the distinct diffusion rates of Cu and Pd during dealloying govern the evolution of surface morphologies. Furthermore, the different chemical activities of Mg and Gd as sacrificial elements, combined with Cu and Pd as major constituents, enable the fabrication of nanoporous surface structures. These combined features make MgCuPdGd an ideal precursor for high‐throughput exploration. Alloy films are prepared via magnetron sputtering, followed by dealloying to generate a nanostructured surface. As a result, distinct micro‐zones with visible color variations are formed, originating from compositional and microstructural gradients introduced during high‐throughput processing. By correlating the nanostructure size in the surface with antimicrobial efficacy, we identify specific size ranges that either promote or inhibit antibacterial activity. This approach allows for the simultaneous screening of both alloy composition and surface structure to optimize antimicrobial performance. Notably, among the tested zones, we successfully screened a sample with superior antimicrobial activity. This study presents a high‐throughput, colorimetric screening strategy that is not limited to antimicrobial applications but can also be extended to a broad range of functional material systems, providing valuable insights for materials design and optimization.

## 2 Results and Discussion

### 2.1 High‐Throughput Synthesis and Characterization

**Figure 1:**
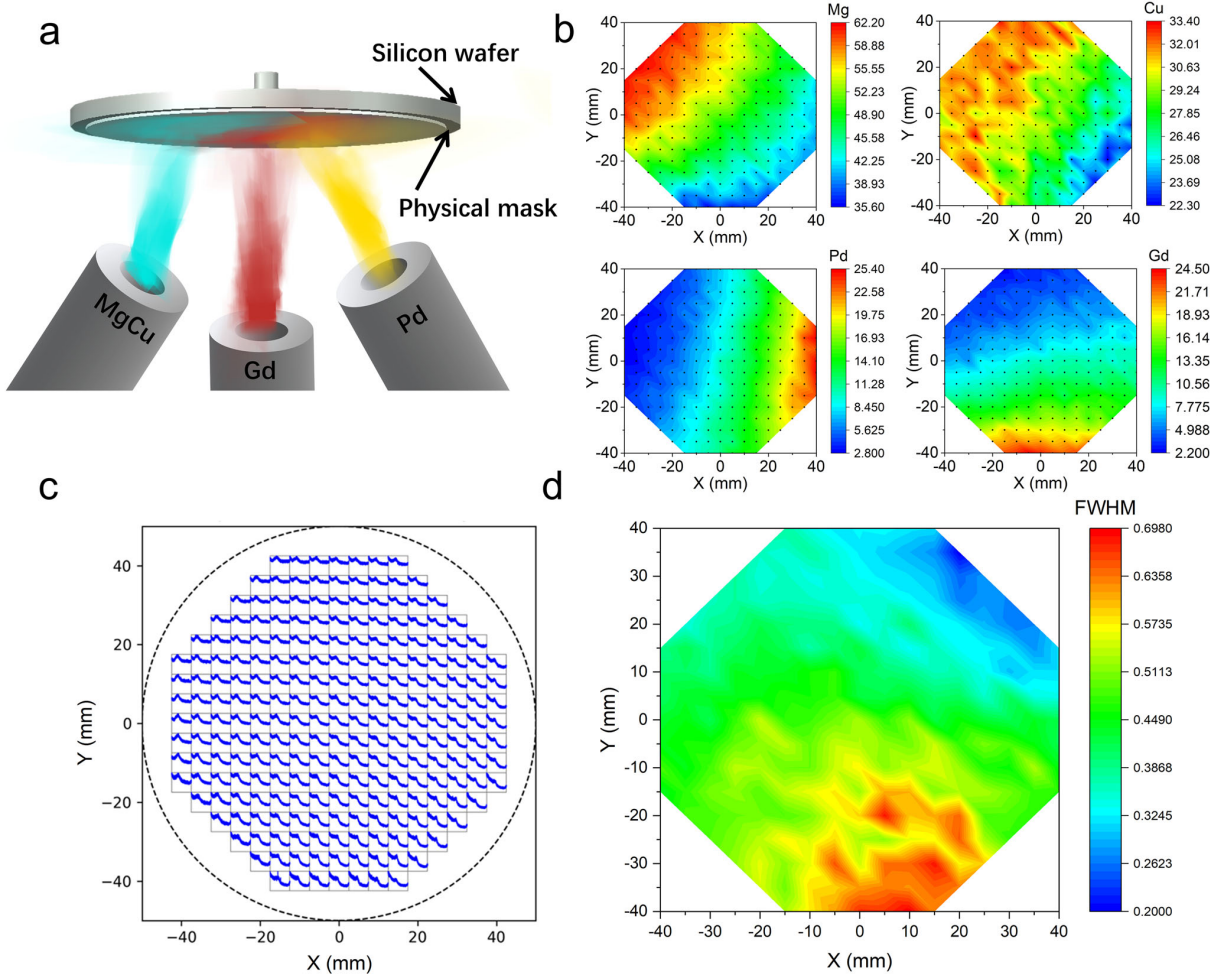
a) Schematic illustration of the alloy composition library fabricated by magnetron sputtering. b) 2D EDS mapping distributions of Mg, Cu, Pd, and Gd for the atomic ratio. c) XRD patterns of the MgCuPdGd alloy library. d) Distribution of the peak width (Δ*q*) of the first diffraction peak across the MgCuPdGd alloy library.

A compositionally graded alloy library was synthesized via magnetron co‐sputtering using three targets: MgCu, Pd, and Gd. In this library system, Mg and Gd act as sacrificial elements to facilitate subsequent dealloying on account of their relatively high chemical activity, while Cu and Pd serve as the primary components due to the strong antibacterial properties and the difference in the intrinsic properties of Cu and Pd, which control the evaluation of morphology during dealloying.^[^ ^32^, ^33^ ^]^ The compositional gradient of the alloy library was controlled by adjusting the tilt angles of each target during deposition (**Figure** **1**a). The resulting composition distribution was analyzed using high‐throughput energy‐dispersive X‐ray spectroscopy (EDS), as shown in Figure 1b and Figure S1 (Supporting Information). The atomic fractions of the elements varied across the library, with Mg ranging from 35.6 to 62.2 at. %, Cu from 22.3 to 33.4 at. %, Pd from 2.8 to 25.4 at. %, and Gd from 2.2 to 24.5 at. %. This broad compositional range enables precise tuning of alloy chemistry, facilitating a systematic investigation of the effects of composition on nanostructure formation, while also enabling exploration of how the Cu‐to‐Pd ratio influences antibacterial performance.

To evaluate the GFA of the alloy library, X‐ray diffraction (XRD) was employed as a rapid and effective screening technique.^[^ ^34^ ^]^ In particular, the full width at half maximum (FWHM, *Δq*) of the first diffraction peak is widely recognized as a practical indicator of GFA, with larger *Δq* values typically corresponding to higher GFA.^[^ ^34^, ^35^ ^]^ Accordingly, high‐resolution micro‐area XRD measurements were conducted on all alloy compositions to extract their respective *Δq* values for GFA evaluation (Figure 1c,d). The results reveal a clear trend: *Δq* values decrease with increasing Pd concentration, indicating that a higher Pd content negatively impacts the GFA of the MgCuPdGd system. Nevertheless, the majority of alloys in the library exhibit *Δq* values ranging from 0.50 to 0.70, confirming that the MgCuPdGd system maintains excellent GFA across a broad compositional window. This robust GFA is particularly advantageous for subsequent dealloying‐based structural and functional screening.

In addition to composition, surface microstructure plays a critical role in determining the antimicrobial properties of metallic materials.^[^ ^36^ ^]^ To enhance surface area and thereby improve antibacterial activity, we conducted high‐throughput chemical dealloying of the MgCuPdGd alloy library to generate tailored nanostructured morphologies. Owing to the relatively homogeneous elemental distribution within each region of the deposited films (Figure S2, Supporting Information), the dealloying process involves not only the selective dissolution of sacrificial elements but also the concurrent diffusion and self‐assembly of more noble metals, resulting in diverse microstructural features.^[^ ^37^ ^]^ Consequently, the resulting morphology is strongly dependent on the local composition of the alloy.

**Figure 2:**
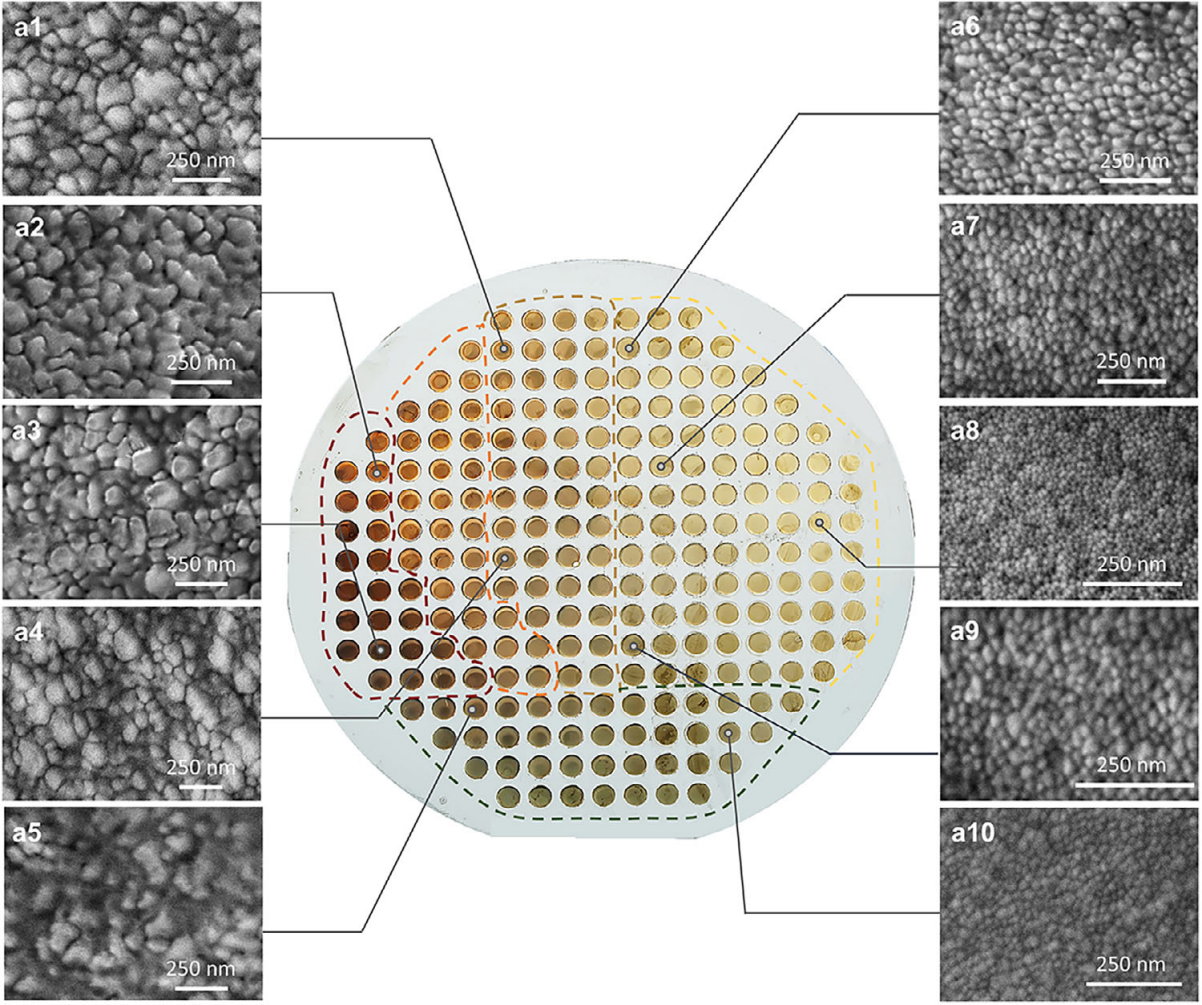
Photographs of all samples after dealloying. (a1–a10): the SEM images of D‐MgCuPdGd in different chromatic zones.

As shown in **Figure** **2**, the dealloyed library (D‐MgCuPdGd) exhibits distinct color gradients‐ranging from red to yellow and green‐corresponding to compositional variations across the library. To validate these visually observed colors, we selected representative samples from distinct color zones (marked by the arrow in Figure S3a, Supporting Information) and conducted reflectance spectroscopy tests using ultraviolet‐visible (UV–vis) spectroscopy (Figure S3b, Supporting Information). The red regions displayed significantly stronger reflectance compared with the overall weaker reflectance of the low‐saturation green regions. Specifically, the D‐MgCuPdGd at (‐40, 10) exhibits a deep red appearance, characterized by pronounced bands near ≈584 and ≈670 nm and a weak feature at ≈720 nm associated with the deep‐red tint. At (−10, 20), a red‐orange color is observed: the ≈720 nm peak disappears while peaks remain near ≈580 and ≈662 nm. At (35, 15), yellow becomes dominant as the ≈670 nm red band disappears and the main features shift to shorter wavelengths. With increasing Gd content, the overall reflectance diminishes and a broad maximum emerges around ≈500 nm, consistent with a dark‐green appearance. To exclude oxidation artifacts, we examined the oxygen content in different regions by EDS (Figure S4, Supporting Information), and found comparable O levels throughout, indicating that the observed color differences do not arise from differential oxidation. We thus attribute the color zoning to intrinsic composition–nanostructure coupling rather than extrinsic surface oxidation; furthermore, synchronized processing of the array minimizes any residual oxidation‐related bias.

Based on visual inspection, the dealloyed samples can be categorized into several regions (Figure 2), each corresponding to distinct compositions and surface morphologies. Scanning electron microscopy (SEM) and EDS were used to investigate these regions and elucidate the correlation among composition, color, and surface structure of D‐MgCuPdGd. Figure 2a1–a5 features a representative nanostructure, in which the ligament size gradually increases from 55 to 120 nm as the color transitions from light brown to dark red. As the color changes from red to yellow, the Cu content decreases gradually, while the Pd content increases (Figure 2a6–2a10). This compositional change plays a critical role in controlling the size of the nanostructure. Notably, Pd has a slower diffusion rate than Cu in the Cu‐Pd alloy during the entire dealloying process, which leads to a reduction in surface nanostructure size.^[^ ^38^, ^39^ ^]^ As a result, the residual elements tend to aggregate into isolated islands that subsequently evolve into fine ligaments. Notably, the green region corresponds to the Gd‐rich region, in which the surface shows very limited porosity and only remnants of ligaments are present.

To validate the feasibility of morphology prediction based on color variation, we analyzed samples with similar hues from the green, yellow, and red regions (Figure S5, Supporting Information). The results confirm that samples with similar colors exhibit comparable surface morphologies. This finding enables predictive identification of material structures based on color similarity across different regions of the library, minimizing the need for exhaustive characterization of each individual sample. Overall, this parallel chromatic‐zone screening strategy significantly reduces time and labor costs associated with high‐throughput structural screening.

### 2.2 Antibacterial Activity Assessment

The antimicrobial activity of metallic materials is primarily governed by their composition, which determines the release of metal ions capable of disrupting bacterial membranes or deactivating proteins.^[^ ^29^ ^]^ However, surface structure also plays a crucial role in modulating antibacterial efficiency.^[^ ^14^ ^]^ Specifically, surface morphology influences bacterial adhesion and contributes to sterilization through physical interactions. For example, nanostructures such as needle‐like or porous features can mechanically disrupt bacterial cell walls, thereby boosting antibacterial performance. Metallic nanoparticles with high surface energy tend to strongly adsorb onto bacterial surfaces, leading to disruption or perforation of the cell membrane.^[^ ^40^, ^41^, ^42^ ^]^ Given that both composition and surface structure synergistically dictate antimicrobial behavior, the rational design of antibacterial materials must involve the simultaneous optimization of these two factors.^[^ ^43^ ^]^ The above high‐throughput screening strategy integrating compositional tuning with surface structure control via magnetron sputtering and chemical dealloying enables the rapid and efficient identification of promising antibacterial candidates.

**Figure 3:**
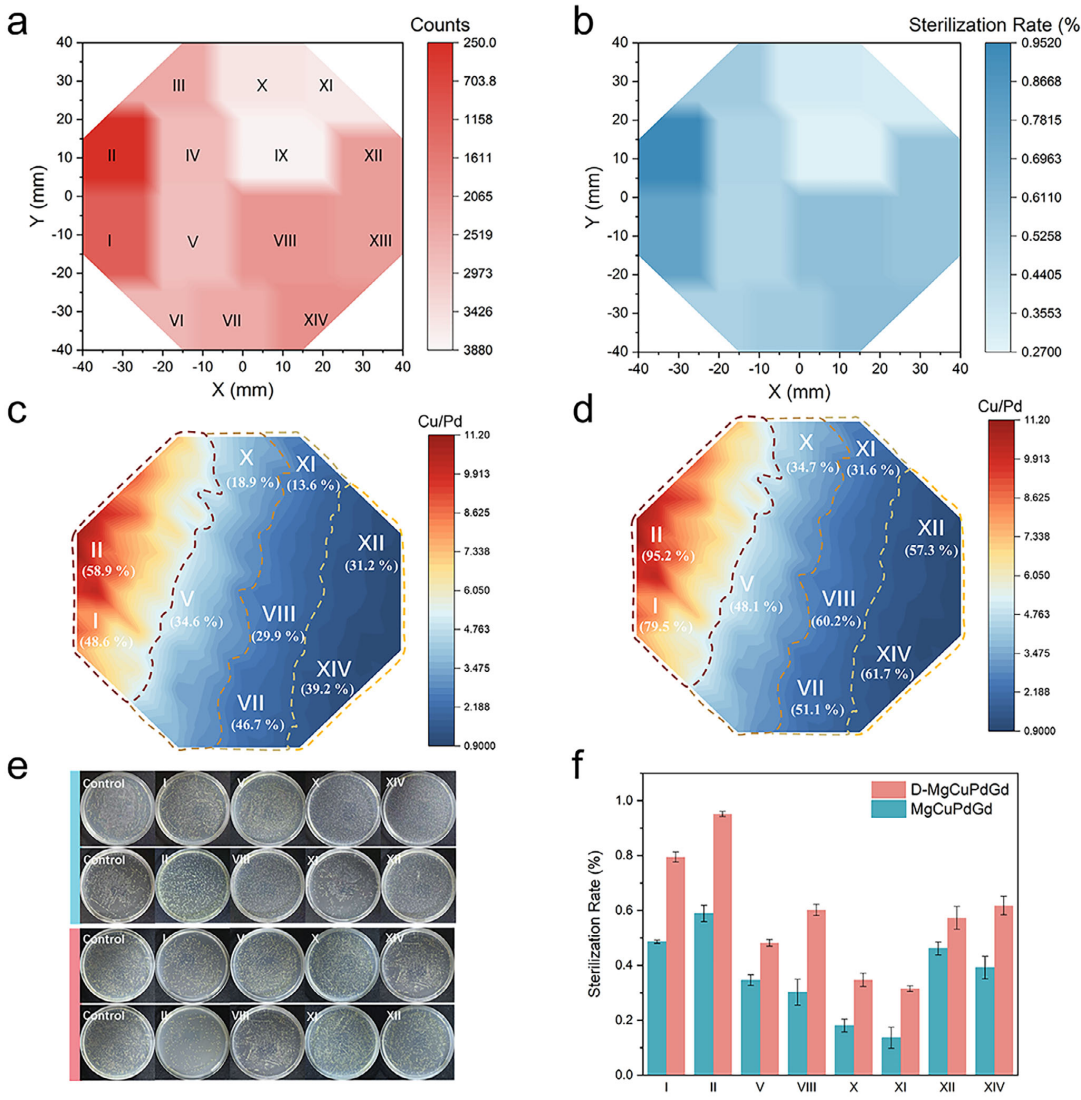
a) Statistical analysis of bacterial colony counts on the D‐MgCuPdGd alloy library after 18 h of co‐culture with bacteria. b) Sterilization rate of the D‐MgCuPdGd alloy library after 24 h of bacterial co‐culture. c) Antimicrobial efficiency map of MgCuPdGd alloy films without surface nanostructures as a function of the Cu/Pd atom ratio. d) Antimicrobial efficiency map of D‐MgCuPdGd alloy films as a function of the Cu/Pd atom ratio Cu/Pd atom ratio. e) Representative images of *S. aureus* colonies grown on the blank control and on Zones I, II, V, VIII, X, XI, XII, and XIV of the D‐MgCuPdGd alloy (red) and corresponding composition films without dealloying treatment (blue). f) Comparison of the antimicrobial properties between dealloyed and non‐dealloyed samples.

The antimicrobial property of the D‐MgCuPdGd library was evaluated against *Staphylococcus aureus* (*S. aureus*). The library was segmented into equal zones based on compositional and morphological variations (Figure 1b and Figure 2) in well plates to test next. **Figure** **3**a,b, and Figures S6 and S7 (Supporting Information) present the residual bacterial counts and the corresponding sterilization efficiencies across different zones. Notably, samples from the red region (Zones I and II), which contain the highest Cu content, exhibited the strongest antibacterial activity, achieving a 95% reduction in viable *S. aureus* cells. In contrast, although Zone X also contains a relatively high Cu concentration, its antibacterial efficiency was significantly lower at only 32.8%. A comparison of their surface morphologies (Figure 2a2–2a6) reveals marked structural differences, indicating that Cu content alone is insufficient to ensure effective antibacterial performance. Instead, surface architecture also plays a critical role. Besides, Zones IX and XI, which exhibit surface morphologies similar to that of Zone X, also exhibit relatively poor antibacterial activity. This suggests that such surface structures may hinder effective contact between bacterial cells and the material surface, thereby compromising antibacterial efficacy. As the Pd content increases, the Pd‐rich zones in the yellow region (Zones XII and XIII) exhibit ≈60% antibacterial efficiency, highlighting the significant antimicrobial contribution of Pd.^[^ ^44^, ^45^ ^]^ Collectively, these results underscore that both elemental composition and surface morphology contribute synergistically to the overall antibacterial performance of the materials.

To further elucidate the key factors governing the antibacterial performance of D‐MgCuPdGd alloys, multiple representative zones are selected from the compositional library for comparative analysis against their as‐deposited counterparts lacking surface nanostructures (MgCuPdGd library) (Figure 3c,d). In all cases, samples lacking surface features display markedly lower antibacterial efficiency than their nanostructured counterparts, underscoring the critical role of surface morphology in enhancing bactericidal activity. Ion release is a primary antimicrobial mechanism for metallic systems; therefore, quantifying the dissolution of reactive ions is essential for designing efficient and durable antimicrobials. To assess the antimicrobial properties of D‐MgCuPdGd, we tested its ionic dissolution behavior using the inductively coupled plasma mass spectrometer (ICP‐MS). The results clearly show that the amount of Mg and Gd dissolved is much higher than that of Cu and Pd, due to its weak passivation ability and the lower oxidation potential (−2.37 and −2.28 V) compared with those of Cu (0.34 V) and Pd (0.92 V). More importantly, the ion release rate of the nanostructured film is significantly larger than that of the original film, suggesting the importance of nanostructures for antimicrobial properties (Tables S1 and S2, Supporting Information). Comparative analysis reveals that regions with higher Cu content, such as Zones I and II, exhibit superior antibacterial activity. Furthermore, increasing the Pd content—particularly when approaching a Cu: Pd ratio of 1:1 (e.g., Zones XII and XIV)—led to further enhancement in antibacterial performance. Gd incorporation also contributes positively: within the blue compositional regime, where Cu/Pd ≈ 1 (Figure 3c,d), antimicrobial activity improves monotonically with Gd content.^[^ ^46^, ^47^ ^]^ This trend is corroborated by the measured Gd ion release, which provides strong evidence for a composition‐activity correlation (Figure S8, Supporting Information). To systematically assess the impact of surface nanostructure, we compare dealloyed (D‐MgCuPdGd) and undealloyed (MgCuPdGd) samples (Figure 3e,f). Notably, the degree of performance enhancement varied among zones, suggesting a structure–activity relationship tied to nanoscale surface characteristics.

**Figure 4:**
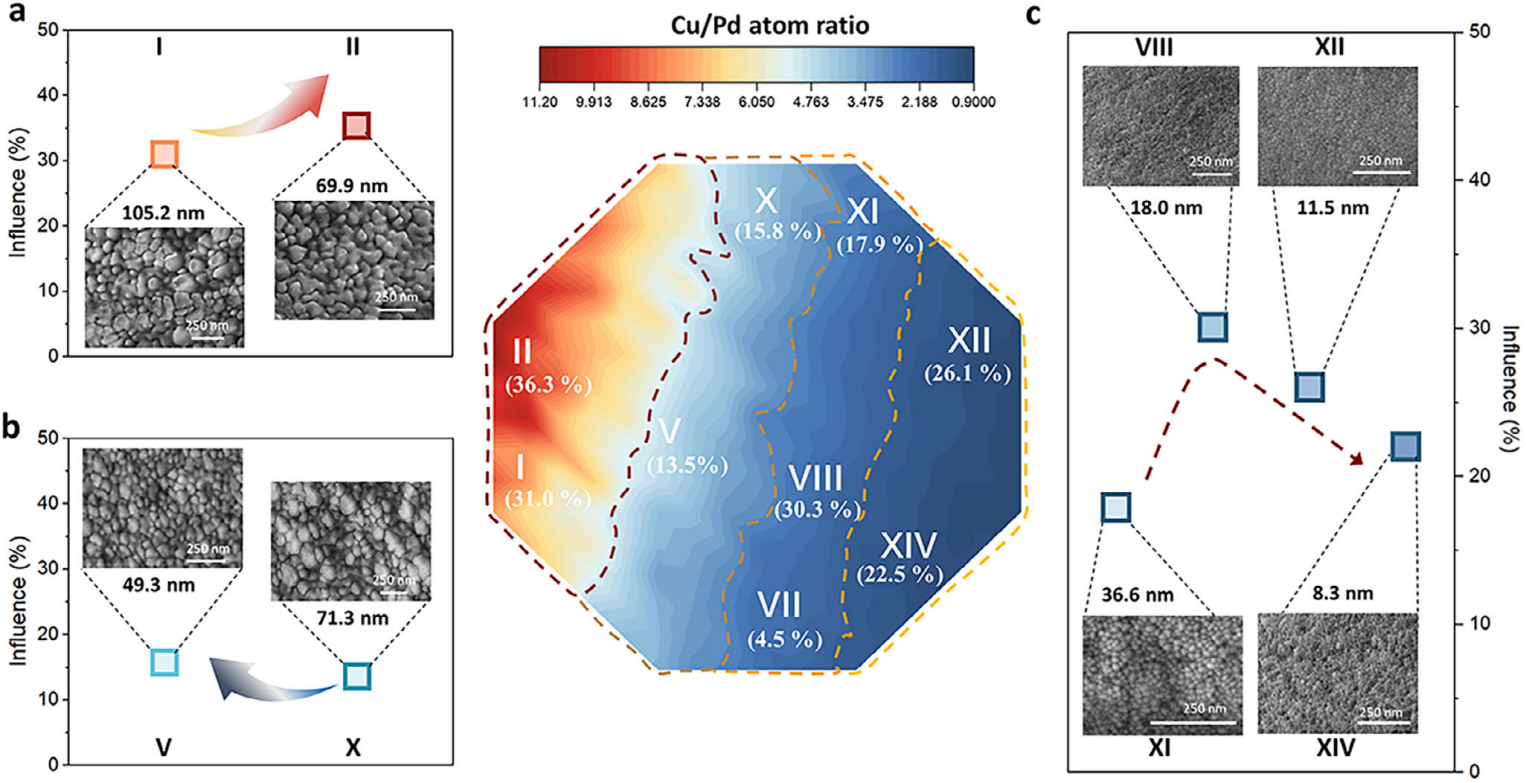
a–c) Statistical analysis of ligament sizes in representative zones and their correlation with antibacterial performance. The red line zone, brown line region, and orange and yellow regions are shown in a, b, and c, respectively. Insets: corresponding SEM images illustrating variations in ligament size across different regions.

To identify the optimal synergy between composition and morphology, we quantified the enhancement in antibacterial activity of D‐MgCuPdGd samples relative to their MgCuPdGd counterparts, and systematically summarized the nanostructure types, characteristic ligament sizes, and corresponding antibacterial efficiencies across selected regions (**Figure** **4**). In the red region where Cu content is significantly higher than that of Pd, the surface exhibits a bicontinuous nanoporous architecture, which shows a strong correlation with improved antibacterial activity (Figure 4a). For instance, in Zone II, the dealloyed film with a bicontinuous nanoporous structure achieves a 36.3% higher antibacterial efficiency compared to its undealloyed counterpart. Even when the ligament size increases to 105 nm (Figure S9, Supporting Information), the nanoporous structure still contributes to an ≈31% improvement in antibacterial performance, highlighting the synergistic effect between surface porosity and chemical composition.

In contrast, the brown compositional region, where the Cu content is relatively reduced, exhibits a surface morphology composed of aggregated nanoparticles with heterogeneous sizes. As shown in Figure 4b and Figure S10 (Supporting Information), these particle‐like surface features are associated with substantially lower antibacterial efficiencies compared to the bicontinuous nanoporous architecture. Similarly, Zone VII, characterized by poorly developed surface textures, exhibits only moderate improvement in antibacterial performance. These observations further underscore the critical role of well‐defined and continuous surface nanostructures in enhancing antibacterial activity.

In the yellow dealloyed region (Figure 2), a gradual increase in Pd content leads to progressive refinement of the surface ligaments, thereby increasing surface roughness. This enhanced roughness likely enlarges the effective contact area between the material surface and bacterial membranes, thus promoting antibacterial efficacy. Although Zone XI exhibits a surface architecture comparable to that of Zones VIII and XII, its antibacterial performance differs significantly, prompting a deeper investigation into the role of ligament size. To quantify this effect, ligament widths were measured across four representative zones (Figure 4c; Figure S11, Supporting Information). The results reveal that a ligament width of ≈36.5 nm corresponds to ≈17.9% bacterial inhibition, whereas further refinement to ≈18 nm results in a peak inhibition of ≈30%. However, excessive reduction in ligament size leads to a decline in antibacterial performance (Figure 4c), suggesting that moderate surface roughness is more favorable for optimizing bactericidal efficacy.^[^ ^48^, ^49^ ^]^ The SEM images of *S. aureus* implicates the importance of surface structure in antibacterial activity, compared with bacteria in zones II and XIV, where the cell walls are clearly damaged, the bacteria in area X remain relatively complete in shape (Figure S12, Supporting Information).

**Figure 5:**
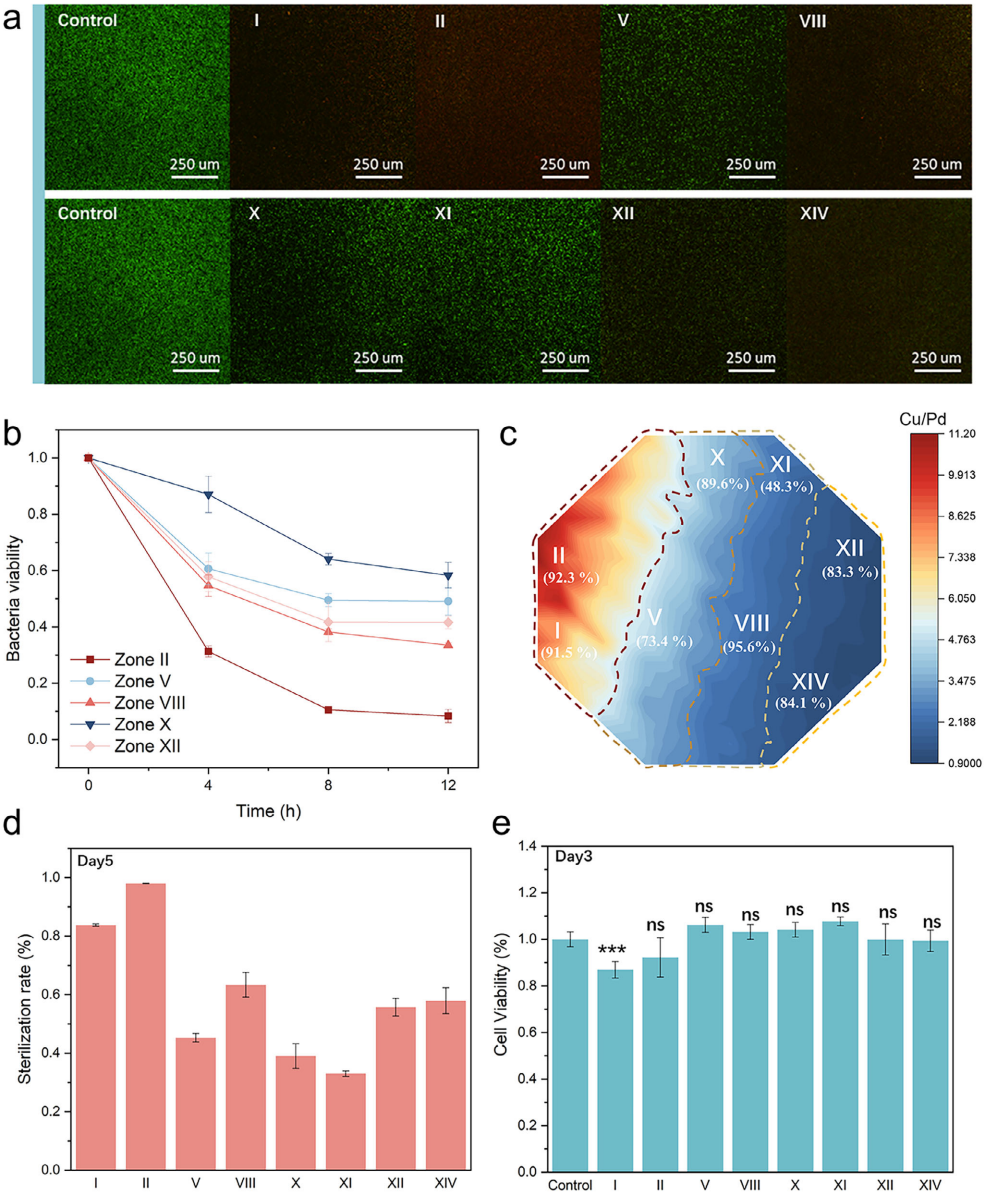
a) Fluorescence images of *S. aureus*. b) Antimicrobial efficiency of D‐MgCuPdGd alloy films after 4, 8, and 12 h of co‐culture with *S. aureus*. c) Antimicrobial efficiency mapping of D‐MgCuPdGd alloy film against *E. coli*. d) Sterilization rate of D‐MgCuPdGd in Zones I, II, V, VIII, X, XI, XII, and XIV for 5 days. e) Cytotoxicity of D‐MgCuPdGd alloy films after 3 days of incubation. ^*^ *p* < 0.05, ^**^ *p*< 0.01, and ^***^ *p* < 0.001.

Next, we thoroughly examined the antibacterial activity and mechanism of the MgCuPdGd thin film. We employed standard live/dead staining of bacteria using SYTO 9 and propidium iodide (PI) probes, wherein PI fluorescence exclusively stains dead bacteria with compromised cell membranes. **Figure** **5**a exhibits large numbers of disrupted bacterial cells with damaged membranes in Zones I, II, and VIII, confirming the antibacterial inhibitory effect of the surface nanostructures and verifying the superior antibacterial efficiency of Zone II. To further assess antibacterial dynamics, we conducted time‐kill assays to continuously monitor bacterial counts. The bacterial growth kinetics results confirm rapid sterilization within a short period as well as a long‐term ability to inhibit bacterial growth (Figure 5b; Figure S13, Supporting Information). To evaluate the broad‐spectrum antibacterial activity, the representative Gram‐negative bacterium (*E. coli*) was chosen for testing. Several representative Zones were selected for antimicrobial evaluation based on previous results (Figure 5c; Figure S14, Supporting Information). The film exhibits significantly enhanced antimicrobial activity. Compared with *S. aureus*, where Zone II (Cu‐rich) demonstrates the best performance, whereas Zone VIII shows the optimal antibacterial activity against *E. coli*, achieving 95.6% antimicrobial efficacy. Consistently, Zone XI exhibits relatively poor performance, whereas zone II also displays strong bactericidal activity, suggesting the versatility of MgCuPdGd alloy films as a broad‐spectrum antimicrobial material.

Long‐term stability is a key indicator for evaluating antimicrobial performance. Therefore, we performed continuous antibacterial tests over five days. Figure 5d demonstrates the sustained efficacy of the film, with Zone II achieving 98% inhibition, confirming its long‐lasting antibacterial capability. An ideal antimicrobial material should also minimize cytotoxicity. Accordingly, we evaluated biocompatibility using a CCK‐8 assay. As shown in Figure 5e and Figure S15 (Supporting Information), after three days of co‐culture with cells, MC3T3 cell viability in each region exceeds 85%, indicating negligible cytotoxicity. These findings further demonstrate the significant application potential of MgCuPdGd alloy films in biological antibacterial applications.

## 3 Conclusion

We propose a combined high‐throughput deposition and parallel chromatic‐zone screening strategy that allows the generation of samples with distinct compositional and microstructural variations, visually distinguished by obvious color differences. This approach minimizes the need for exhaustive characterization of each individual sample and significantly reduces time and labor costs. Specifically, the MgCuPdGd alloy library, comprising 229 samples, exhibits three primary chromatic regions: red, yellow, and green. The red regions are characterized by a bi‐continuous nanoporous structure, the yellow regions exhibit a nanoparticle morphology with smaller ligaments resembling particle aggregation, and the green regions display minimal surface porosity. Notably, the red regions exhibit the highest antibacterial performance, achieving a 95% reduction in *S. aureus* viability. This superior activity is attributed to the synergistic effects of Cu and Pd elements and the optimized nanostructure.

This work presents a novel and efficient methodology for materials development, providing valuable insights and new design principles for the rapid discovery of functional materials with tailored surface structures and enhanced properties. Looking ahead, several directions could further strengthen the impact and applicability of this chromatic‐zone screening strategy. First, expanding the methodology to multiple alloy systems will validate its generalizability and broaden its scope beyond the present case study. Second, clinical validation and multi‐pathogen testing will be pursued to establish real‐world relevance and demonstrate broad‐spectrum antimicrobial performance. Third, integration of this strategy with machine learning algorithms could revolutionize the autonomous mapping of composition–microstructure–activity relationships, enabling real‐time prediction and optimization of antimicrobial performance across vast material libraries. Finally, we envision the development of a color‐oriented, multi‐system database to facilitate reverse engineering of materials. Such a database would allow users to query target performance criteria and directly obtain the corresponding color ranges and preparation parameters, thereby significantly shortening the materials discovery and optimization cycle. Together, these advances will render the chromatic‐zone concept more actionable and accelerate its translation into practical applications.

## 4 Experimental Section

### Materials

All chemicals were of analytical grade. Sulfuric acid (H_2_SO_4_) was purchased from Beijing Tongguang Fine Chemicals Co., Ltd. Sodium chloride (NaCl) and 0.01 m phosphate‐buffered saline (PBS) were obtained from Aladdin. Tryptone was purchased from Thermo, while agar powder and beef extract powder were supplied by Qingdao Hope Bio‐Technology Co., Ltd. Staphylococcus aureus (*S. aureus*) and *Escherichia coli* (*E. coli*) strains were obtained from the Guangdong Microbial Culture Collection Center. Metallic targets, including MgCu, Pd, and Gd, were purchased from Beijing Jiaming Platinum Nonferrous Metals Co., Ltd.

### Synthesis of MgCuPdGd and D‐MgCuPdGd

A composition library containing 229 alloys was prepared by magnetron co‐sputtering technique using three targets with purity higher than 99.9% sputtered under a DC power supply (MgCu (65:35 at. %): 60 W, Gd: 45 W, and Pd: 5 W). The deposition was performed on a 100 mm diameter silicon wafer. A metal shadow mask, made from a 500 µm thick steel plate, was employed to spatially isolate the deposited areas, generating 229 discrete alloy composition points. The compositional gradients across the alloy libraries were controlled by adjusting the sputtering power based on the deposition rate measured with a quartz crystal thickness monitor. Prior to deposition, all targets were pre‐sputtered to remove surface oxides and possible contaminants. During sputtering, the chamber base pressure was maintained below 5 × 10^−4^ Pa, and high‐purity argon was used to sustain a working pressure of 1 Pa. The deposition rates of Pd, Gd, and MgCu targets at powers of 5, 45, and 60 W were 18, 72 and 162 Å min^−1^, measured by a film thickness gauge. The MgCuPdGd thin film was deposited for 3 h. Following deposition, the entire wafer was immersed in 5 mm sulfuric acid (H_2_SO_4_) for chemical dealloying to obtain D‐MgCuPdGd.

### Characterization

The chemical composition and surface morphology of the MgCuPdGd film were analyzed by X‐ray energy spectroscopy (EDS, BRUKER XFlash6160) using field emission scanning electron microscopy (SEM, HITACHI SU5000, 15 keV). The structure characterization was carried out using a Malvern PANalytical Empyrean X‐ray diffraction (XRD) with Cu‐Kα radiation. The resulting XRD spectra were processed and analyzed following the method reported in the literature.^[^ ^35^ ^]^

### Antimicrobial Performance Evaluation–Bacterial Pretreatment

*Staphylococcus aureus* (*S. aureus*) was used to assess the antimicrobial capacity of the samples. The experimental strain *S. aureus* was cultured and maintained using Luria‐Bertani (LB) medium. Before the antibacterial test, frozen *S. aureus* strains were first inoculated into LB liquid medium and incubated in a constant temperature shaker at 37 °C for 24 h for activation. Subsequently, the activated bacterial solution was spread on LB solid medium plates. After the colonies grew, the plates were inverted and stored in the refrigerator at 4 °C for subsequent experiments. Before each experiment, several colonies of *S. aureus* were selected from preserved LB solid medium plates and inoculated into LB liquid medium. The inoculated medium was incubated in a constant temperature shaker at 37 °C for 12 h to allow the bacteria to resuscitate and reach the logarithmic growth phase.

### Antimicrobial Performance Evaluation–Antibacterial Ability of D‐MgCuPdGd and MgCuPdGd alloy library film

The autoclaved metal sample was placed in a sterile 12‐well plate. The shaken bacterial solution was diluted to 0.1 OD with LB liquid medium (OD = 0.1 corresponds to a bacterial concentration of 1.0 × 10^7^ cfu ml^−1^), and then the bacterial solution with OD = 0.1 was diluted by 100‐fold with sterile phosphate‐buffered solution (PBS), which was the bacterial concentration used in the experiment. 50 µL of the above bacterial solution was pipetted onto the surface of the sterilized metal sheet samples and incubated for 24 h at 37 °C in a constant temperature incubator. After incubation, the bacteria attached to the surface of the film were fully eluted into the PBS by vigorous shaking using a vortex oscillator. The resulting bacterial suspension was subjected to a tenfold gradient dilution. Subsequently, 100 µL of the bacterial suspension for each dilution gradient was aspirated and added dropwise to an LB solid medium plate and spread evenly using sterile glass beads. The coated plates were incubated in a constant temperature incubator at 37 °C for 24 h to facilitate bacterial colony growth. Finally, the colonies on each plate were counted, and three parallel experiments were performed for each sample in order to improve the reliability of the experimental results.

(1) $$Antibacterical\;Efficiency=\left(1-\frac{N_{treated}}{N_{control}}\right)\times 100\%$$

where *N*_treated_ and *N*_control_ represent the colony number for the alloy library and the control group, divided by the dilution factor.

*Staphylococcus aureus* (*S. aureus*) and *Escherichia coli* (*E. coli*) were used to assess the antimicrobial capacity of the samples. The experimental strain *S. aureus* was cultured and maintained using Luria‐Bertani (LB) medium. Before the antibacterial test, frozen *S. aureus* strains were first inoculated into LB liquid medium and incubated in a constant temperature shaker at 37 °C for 24 h for activation. Subsequently, the activated bacterial solution was spread on LB solid medium plates. After the colonies grew, the plates were inverted and stored in the refrigerator at 4 °C for subsequent experiments. Before each experiment, several colonies of *S. aureus* were selected from preserved LB solid medium plates and inoculated into LB liquid medium. The inoculated medium was incubated in a constant temperature shaker at 37 °C for 12 h to allow the bacteria to resuscitate and reach the logarithmic growth phase.

### Antimicrobial Performance Evaluation–Bacterial Anti‐Adhesive Assessment

The samples and control were placed separately into conical bottles, into which 10 mL *S. aureus* bacterial suspension with 10^7^ CFU mL^−1^ was added. The flasks were incubated for 4 h at 37 °C in a shaking incubator, allowing bacteria to attach to the sample surfaces. The samples and controls were then transferred into sterilized tubes containing 5 mL PBS, and bacteria were detached from their surface using rotational oscillation in the shaking incubator at 180 rpm for 30 min. Subsequently, 100 µL of bacterial suspension was taken out of the tubes, evenly spread on the plates, and incubated for 24 h at 37 °C.

(2) $$Bacterial\;anti-adhesive\;rate=\left(1-\frac{N_{sample}}{N_{control}}\right)\times 100\%$$

where *N*_treated_ and *N*_control_ represent the colony number for the alloy library and the control group, divided by the dilution factor.

### Antimicrobial Performance Evaluation–The Morphology of Bacteria Adhering to the Composite Surface

Alloy film wafers were placed into a 48‐well plate. Each wafer was covered with 30 µL of co‐culture medium and incubated overnight. The following day, 50 µL of fixative was added to each wafer, and the samples were fixed in the dark for 6 h. After fixation, the wafers were sequentially dehydrated using ethanol solutions of 30%, 50%, 70%, 90%, and 100% (wt.%). After completion, the wafers were mounted and examined by SEM to observe bacterial morphology.

### Antimicrobial Performance Evaluation–Live/Dead Bacterial Viability Assay for Antibacterial and Bacterial Anti‐Adhesive Assessment

In antibacterial and bacterial anti‐adhesive tests, a fluorescence staining method was employed to directly assess bacterial viability. Bacterial suspensions were co‐cultured with the material at 37 °C incubator for 24 h. The mixtures were collected, centrifuged at 5000 rpm for 10 min, the supernatant discarded, and the pellets resuspended in 1 mL physiological saline. The bacteria were stained in the dark for 15 min with the fluorescent dye mixture of green fluorescent dye and red propidium iodide (SYTO‐9/PI). The stained bacteria were observed under a confocal fluorescence microscope (Olympus BX51).

### Antimicrobial Performance Evaluation–In Vitro Cytocompatibility Assay

Extraction solutions were prepared by immersing the material in culture medium for 24 h at 37 °C. MC3T3 cells (2000 cells/well) were seeded in 96‐well plates with 100 µL complete medium. After cell attachment, the medium was replaced with an equal volume of extraction solution. Cells were cultured for 1 and 3 days, and absorbance was measured at each time point to evaluate cell viability.

### Antimicrobial Performance Evaluation–Crystal Violet Staining

*S. aureus* suspension (10^7^ CFU mL^−1^) was mixed with D‐MgCuPdGd and incubated at 37 °C for 72 h. After incubation, the suspension was discarded, and the biofilm was stained with crystal violet (CV) solution for 30 min, followed by PBS washing. The biofilms were fixed with pentanediol and incubated in the dark for 4–6 h, then restrained with 0.01% CV solution for 15 min. Excess stain was removed by PBS washing. The stained biofilms were photographed. Subsequently, 30% acetic acid was added, allowed to stand for 30 min, and the OD value at 590 nm was measured using a microplate reader.

### Antimicrobial Performance Evaluation–Statistical Analysis

All experiments were performed with at least three independent replications and analyzed using Origin 2021, GraphPad Prism 10.1.2, and Nano Measure 1.2 software. The results were expressed as mean ± standard deviations (SD). Statistical analyses were assessed using one‐way analysis of variance (ANOVA). Significance levels were denoted as *p* < 0.05 (^*^), *p* < 0.01 (^**^), and *p* < 0.001 (^***^). A *p*‐value of less than 0.05 was considered statistically significant.

## Conflict of Interest

The authors declare no conflict of interest.

## Supporting information



Supporting Information

## Data Availability

The data that support the findings of this study are available from the corresponding author upon reasonable request.

## References

[1] J. A. Lemire , J. J. Harrison , R. J. Turner , Nat. Rev. Microbiol. 2013, 11, 371. 23669886 10.1038/nrmicro3028

[2] D. Ferber , Science 2003, 301, 1027. 12933980 10.1126/science.301.5636.1027

[3] J.‐J. Zheng , X. Wang , Z. Li , X. Shen , G. Wei , P. Xia , Y.‐G. Zhou , H. Wei , X. Gao , ACS Nano 2024, 18, 1531. 38164912 10.1021/acsnano.3c09128

[4] S. Bagcchi , Lancet Microbe 2023, 4, 20. 10.1016/S2666-5247(23)00119-2 37148896

[5] A. Frei , A. D. Verderosa , A. G. Elliott , J. Zuegg , M. A. T. Blaskovich , Nat. Rev. Chem. 2023, 7, 202. 37117903 10.1038/s41570-023-00463-4 PMC9907218

[6] R. Lv , Y.‐Q. Liang , Z.‐Y. Li , S.‐L. Zhu , Z.‐D. Cui , S.‐L. Wu , Rare Met. 2021, 41, 639.

[7] J. L. Martinez , Environ. Pollut. 2009, 157, 2893. 19560847 10.1016/j.envpol.2009.05.051

[8] Y. Zheng , Y. Luo , K. Feng , W. Zhang , G. Chen , ACS Macro Lett. 2019, 8, 326. 35650837 10.1021/acsmacrolett.9b00091

[9] C. Wang , S. Zhu , Y. Liang , C. Qin , F. Wang , H. Wang , C. Chang , A. Inoue , J. Colloid Interf. Sci. 2023, 645, 287. 10.1016/j.jcis.2023.04.153 37150002

[10] Y. Wang , Y. Yang , Y. Shi , H. Song , C. Yu , Adv. Mater. 2020, 32, 1904106. 10.1002/adma.201904106 31799752

[11] Y. Liu , J. Chen , J. Yang , J. Chen , Q. Hao , J. Guo , Y. Yang , J. Liu , X. Sun , Nanoscale 2025, 17, 14441. 40439153 10.1039/d5nr00406c

[12] S. Stankic , S. Suman , F. Haque , J. Vidic , J. Nanobiotechnol 2016, 14, 73. 10.1186/s12951-016-0225-6 PMC5075760 27776555

[13] C. Wang , X. Wei , L. Zhong , C.‐L. Chan , H. Li , H. Sun , J. Am. Chem. Soc. 2025, 147, 12361. 40063057 10.1021/jacs.4c16035 PMC12007004

[14] P. Makvandi , C. Y. Wang , E. N. Zare , A. Borzacchiello , L. n. Niu , F. R. Tay , Adv. Funct. Mater. 2020, 30, 1910021.

[15] S. A. Anuj , H. P. Gajera , D. G. Hirpara , B. A. Golakiya , J. Trace Elem. Med. Bios 2019, 51, 219. 10.1016/j.jtemb.2018.04.028 PMC7126441 29735327

[16] Y. Qiao , F. Ma , C. Liu , B. Zhou , Q. Wei , W. Li , D. Zhong , Y. Li , M. Zhou , ACS Appl. Mater. Interfaces 2018, 10, 193. 29215863 10.1021/acsami.7b15251

[17] N. A. Al‐Dhabi , A.‐K. Mohammed Ghilan , M. V. Arasu , Nanomaterials 2018, 8, 279. 29701657 10.3390/nano8050279 PMC5977293

[18] Q. Li , S. Zhang , Y. Xu , Y. Guo , Y. Liu , Adv. Sci. 2025, 12, 2501327. 10.1002/advs.202501327 PMC12224937 40285557

[19] M. Hakamada , S. Taniguchi , M. Mabuchi , J. Mater. Res. 2017, 32, 1787.

[20] C. Zhang , X. Wang , J. Sun , T. Kou , Z. Zhang , CrystEngComm 2013, 15, 3965.

[21] S. Chirra , S. Siliveri , R. Gangalla , S. Goskula , S. R. Gujjula , A. K. Adepu , R. Anumula , S. S. Sivasoorian , L.‐F. Wang , V. Narayanan , J. Mater. Chem. B 2019, 7, 7235. 31664291 10.1039/c9tb01736d

[22] M. Birkett , L. Dover , C. Cherian Lukose , A. W. Zia , M. M. Tambuwala , Á. Serrano‐Aroca , Int. J. Mol. Sci. 2022, 23, 1162. 35163084 10.3390/ijms23031162 PMC8835042

[23] H. Peng , D. Dong , S. Feng , Y. Guo , J. Yu , C. Gan , X. Hu , Z. Qin , Y. Liu , Y. Gao , Chem. Eng. J. 2025, 507, 160726.

[24] J. Cao , X. Jiang , Q. Zhang , F. Yuan , J. Yu , F. Yang , M. Li , C. Wang , Y. Lu , M. Li , W. Wang , Y. Liu , Mater. Futures 2023, 2, 025002.

[25] K. Wieczerzak , F. F. Klimashin , A. Sharma , S. Altenried , K. Maniura‐Weber , Q. Ren , J. Michler , ACS Appl. Mater. Interfaces 2024, 16, 60018. 39453916 10.1021/acsami.4c14689 PMC11551899

[26] Z. Wang , J. Liu , C. Qin , H. Yu , X. Xia , C. Wang , Y. Zhang , Q. Hu , W. Zhao , Nanomaterials 2015, 5, 697. 28347030 10.3390/nano5020697 PMC5312890

[27] Z. Zhang , C. Wang , P. Liu , K. M. Reddy , X. Wang , M. Chen , S. Song , Int. J. Plasticity 2022, 152, 103232.

[28] M. Li , J. Liu , C. Wang , Y. Liu , Y. Sun , C. Qin , Z. Wang , Y. Li , L. Liu , S. Liu , Chem. Eng. J. 2022, 427, 130861.

[29] Y. Li , Y. Miao , L. Yang , Y. Zhao , K. Wu , Z. Lu , Z. Hu , J. Guo , Adv. Sci. 2022, 9, 2202684. 10.1002/advs.202202684 PMC9507365 35876402

[30] G. H. Kim , T. An , G. Lim , ACS Appl. Mater. Interfaces 2017, 9, 19057. 28530389 10.1021/acsami.6b15892

[31] D. Wang , Z. Liu , H. Wang , M. Li , L. J. Guo , C. Zhang , Nanophotonics 2023, 12, 1019. 39634932 10.1515/nanoph-2022-0063 PMC11501295

[32] F. Ameen , H. Karimi‐Maleh , R. Darabi , M. Akin , A. Ayati , S. Ayyildiz , M. Bekmezci , R. Bayat , F. Sen , Environ. Res. 2023, 221, 115287. 36640937 10.1016/j.envres.2023.115287

[33] K. Y. Kwon , S. Cheeseman , A. Frias‐De‐Diego , H. Hong , J. Yang , W. Jung , H. Yin , B. J. Murdoch , F. Scholle , N. Crook , E. Crisci , M. D. Dickey , V. K. Truong , T.‐i. Kim , Adv. Mater. 2021, 33, 2104298. 10.1002/adma.202104298 34550628

[34] M.‐X. Li , Y. Sun , C. Wang , L. Hu , S. Sohn , J. Schroers , W. Wang , Y. Liu , Nat. Mater. 2021, 21, 165. 34737454 10.1038/s41563-021-01129-6

[35] S. Ding , Y. Liu , Y. Li , Z. Liu , S. Sohn , F. J. Walker , J. Schroers , Nat. Mater. 2014, 13, 494. 24728462 10.1038/nmat3939

[36] H. Xin , Y. Liu , Y. Xiao , M. Wen , L. Sheng , Z. Jia , Adv. Funct. Mater. 2024, 34, 2402607.

[37] J. Erlebacher , M. Aziz , A. Karma , N. Dimitrov , K. Sieradzki , Nature 2001, 410, 450. 11260708 10.1038/35068529

[38] Z. Zhang , Y. Wang , Z. Qi , W. Zhang , J. Qin , J. Frenzel , J. Phys. Chem. C 2009, 113, 12629.

[39] I. McCue , A. Karma , J. Erlebacher , MRS Bull. 2018, 43, 27.

[40] S. Tang , J. Zheng , Adv. Healthcare Mater. 2018, 7, 1701503.

[41] K. Y. Gudz , E. S. Permyakova , A. T. Matveev , A. V. Bondarev , A. M. Manakhov , D. A. Sidorenko , S. Y. Filippovich , A. V. Brouchkov , D. V. Golberg , S. G. Ignatov , D. V. Shtansky , ACS Appl. Mater. Interfaces 2020, 12, 42485. 32845601 10.1021/acsami.0c10169

[42] S. Wu , F. Zuber , J. Brugger , K. Maniura‐Weber , Q. Ren , Nanoscale 2016, 8, 2620. 26648134 10.1039/c5nr06157a

[43] J. Hou , Y. Xianyu , Small 2023, 19, 2302640. 10.1002/smll.202302640 37322391

[44] F. Meng , X. Qin , L. Yang , F. Huang , J. Diao , X. Cai , D. Zhang , L. Li , P. Zhu , M. Peng , N. Wang , D. Xiao , L. Xia , H. Liu , D. Ma , Small 2022, 18, 2203283. 10.1002/smll.202203283 35871548

[45] G. Fang , W. Li , X. Shen , J. M. Perez‐Aguilar , Y. Chong , X. Gao , Z. Chai , C. Chen , C. Ge , R. Zhou , Nat. Commun. 2018, 9, 129. 29317632 10.1038/s41467-017-02502-3 PMC5760645

[46] Y. Yu , T. Cui , C. Liu , W. Yang , B. Zhang , Adv. Sci. 2025, 12, 2415209. 10.1002/advs.202415209 PMC12005816 39976077

[47] K. D. Khalil , A. H. Bashal , T. Habeeb , A. M. Abu‐Dief , Appl.Organomet. Chem. 2024, 38, 7531.

[48] S. M. Dizaj , F. Lotfipour , M. Barzegar‐Jalali , M. H. Zarrintan , K. Adibkia , Mat. Sci. Eng. C‐Mater. 2014, 44, 278. 10.1016/j.msec.2014.08.031 25280707

[49] X. Zhang , C. Yang , T. Xi , J. Zhao , K. Yang , ACS Appl. Mater. Interfaces 2021, 13, 2303. 33395246 10.1021/acsami.0c19655

