# Supplementary material for: Chromatic‐Zone Mapping for Rapid Discovery of Antibacterial Alloys with Nanostructured Surfaces
Source: Adv Sci (Weinh). 2025 Oct 24;13(3):e13454. doi: 10.1002/advs.202513454 (PMC12806383; doi:10.1002/advs.202513454)
Supplement: Supplementary file 1 — Supporting Information [file ADVS-13-e13454-s001.docx]

Supporting Information

**Chromatic-Zone Mapping for Rapid Discovery of** **Antibacterial Nanoporous Alloys**

*Qiu-Yu Zhao^1,3^, Yu-Ying Liu^2^, Li-Wei Hu^1^, Hong-Xi Duan^1^, Ming-Xing Li^1^, Yan-Hui Liu^1^, Jing Jiang^2^*, Zhen Lu^1,4^*, Wei-Hua Wang^1,5^*

^1^Institute of Physics, Chinese Academy of Sciences, Beijing 100190, China.

^2^School of Health Sciences & Biomedical Engineering, Hebei University of Technology, Tianjin 300130, China.

^3^College of Materials Science and Optoelectronic Technology, University of Chinese Academy of Sciences, Beijing 100049, China.

^4^School of Physical Science, University of Chinese Academy of Sciences, Beijing 100049, China.

^5^Songshan Lake Materials Laboratory, Dongguan, Guangdong 523808, China.


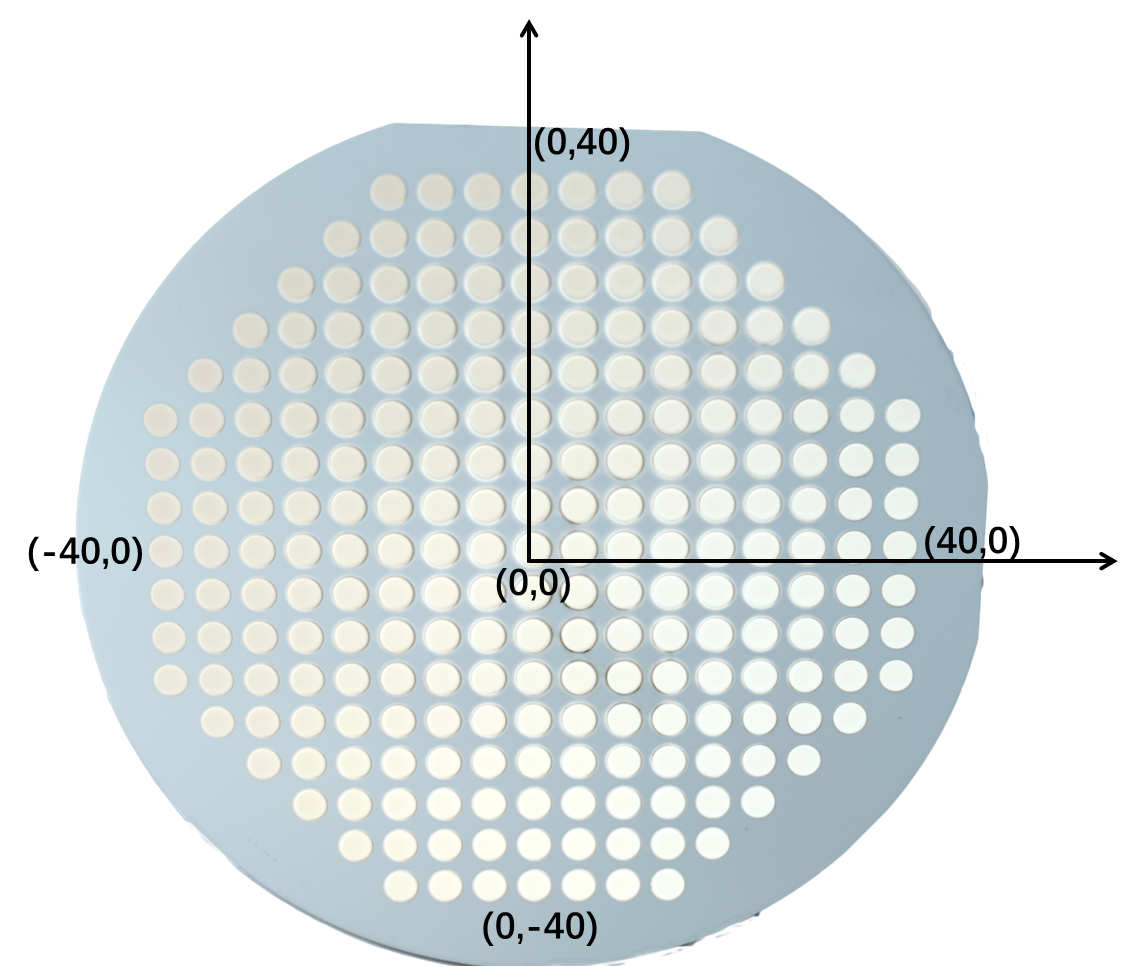


**Figure S1** Photographs and coordinates of each sample in the MgCuPdGd alloy library.
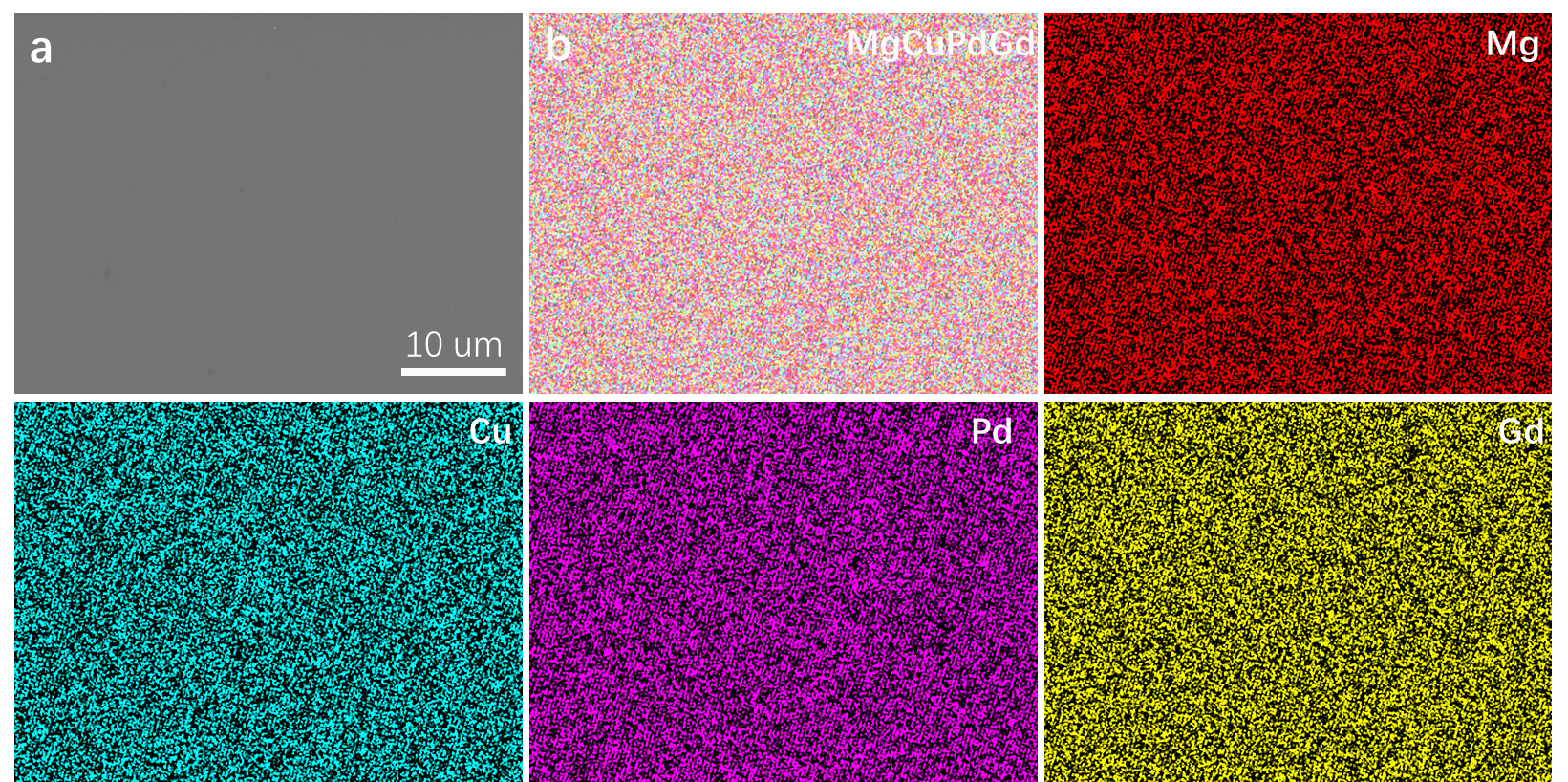


**Figure S2** SEM images and corresponding EDS mappings of MgCuPdGd alloy.


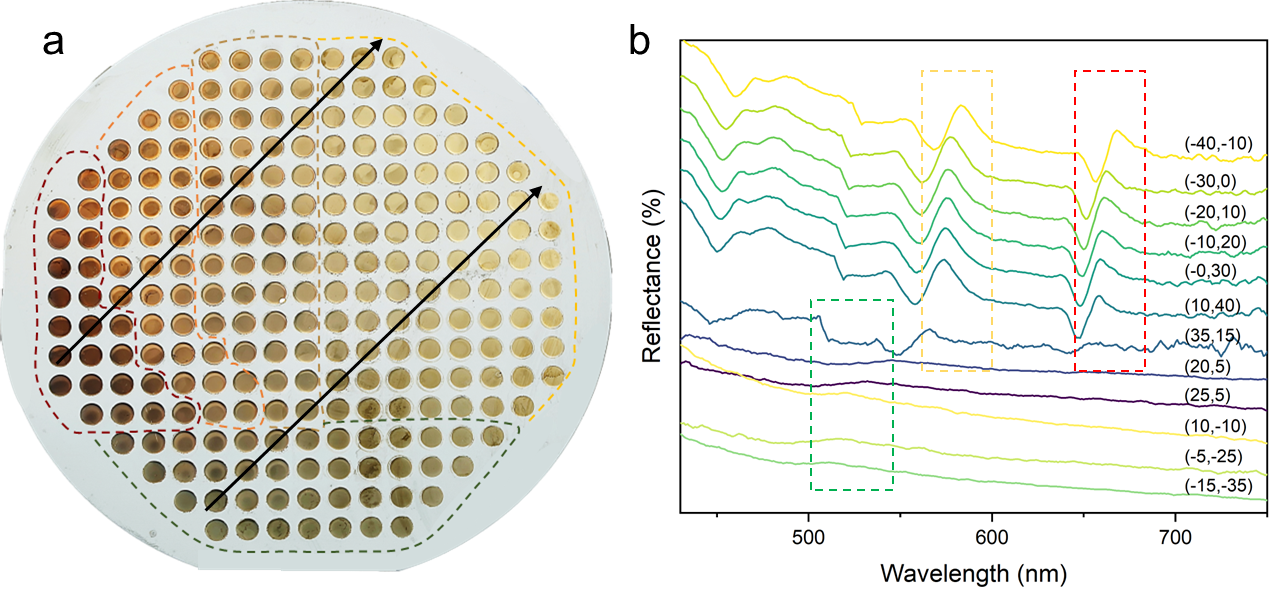


**Figure S3** (a) Photograph of the D-MgCuPdGd alloy film, with the arrow indicating the selected composition. (b) Corresponding reflectance spectra in the visible light range.


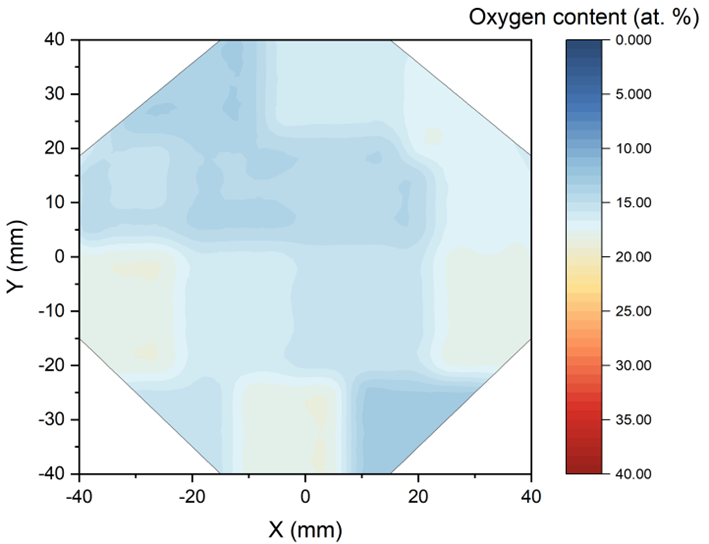


**Figure S4** Oxygen elemental mapping of the D-MgCuPdGd alloy film.


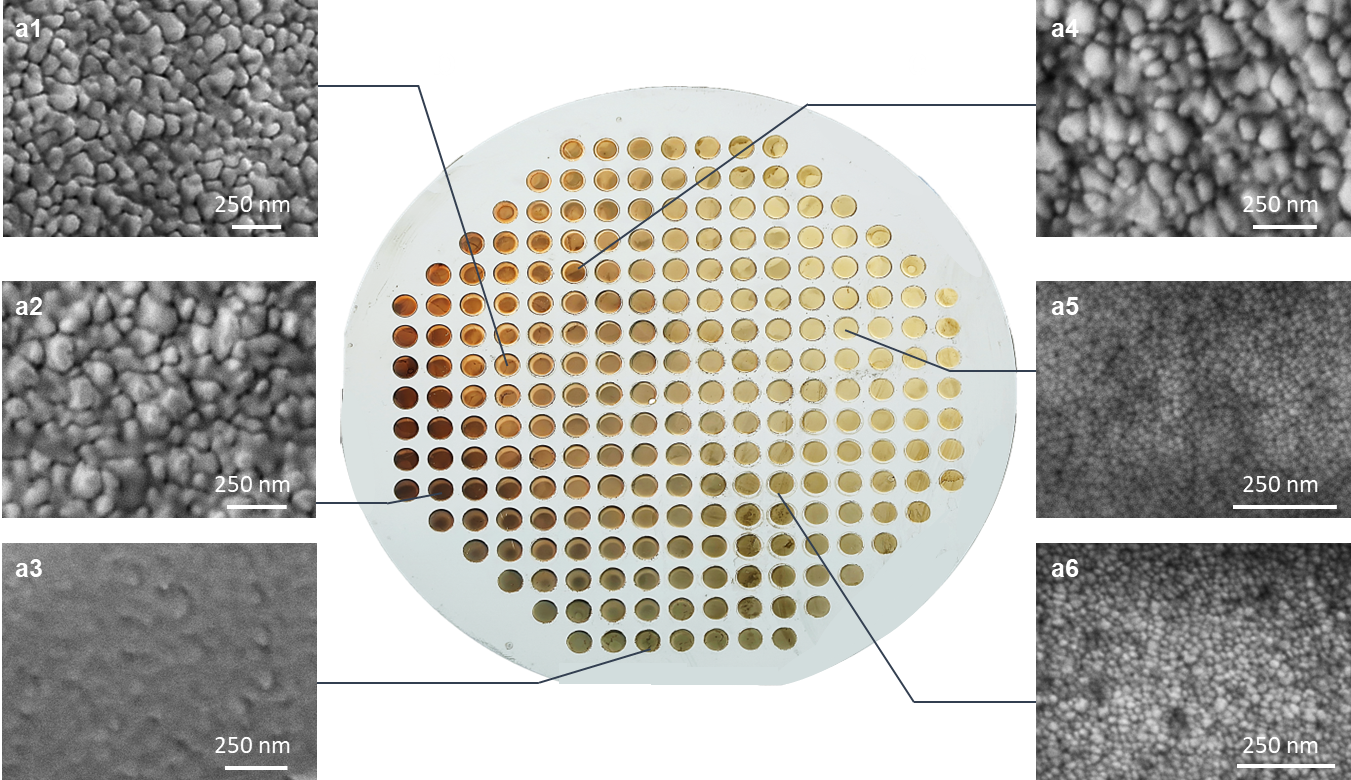


**Figure S5** SEM images at different locations.


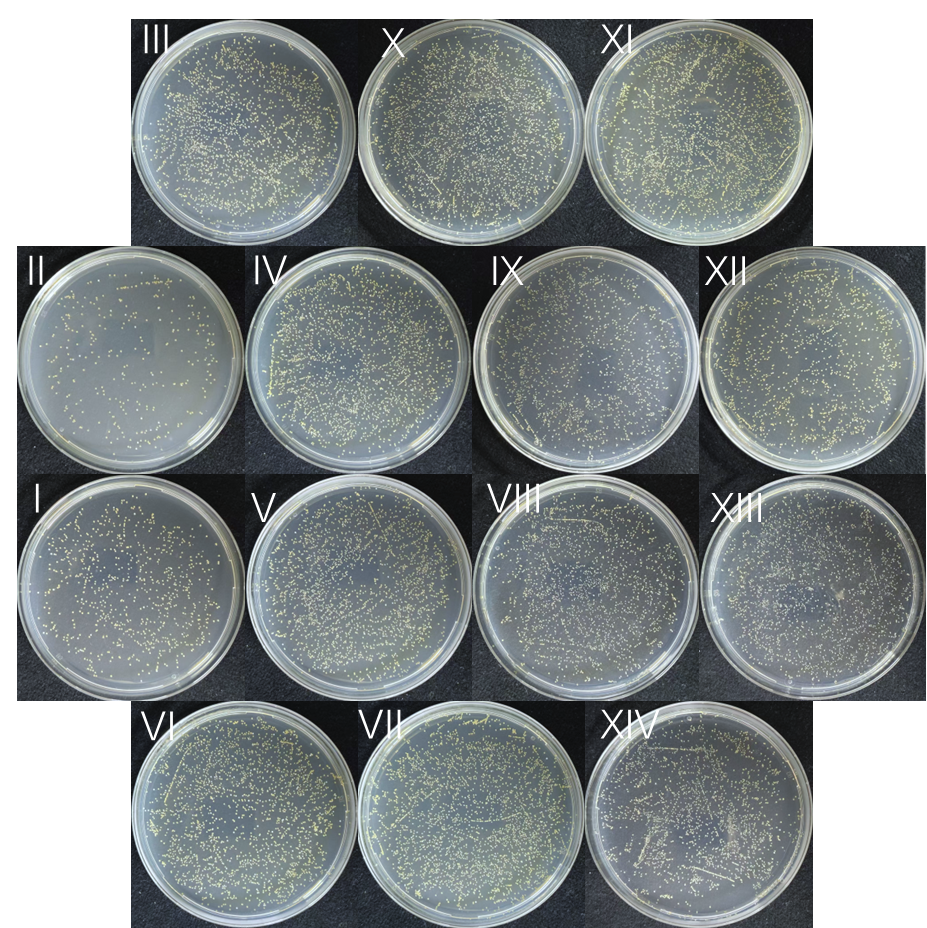


**Figure S6** Colony images of *S. aureus* in different zones of D-MgCuPdGd alloy films.


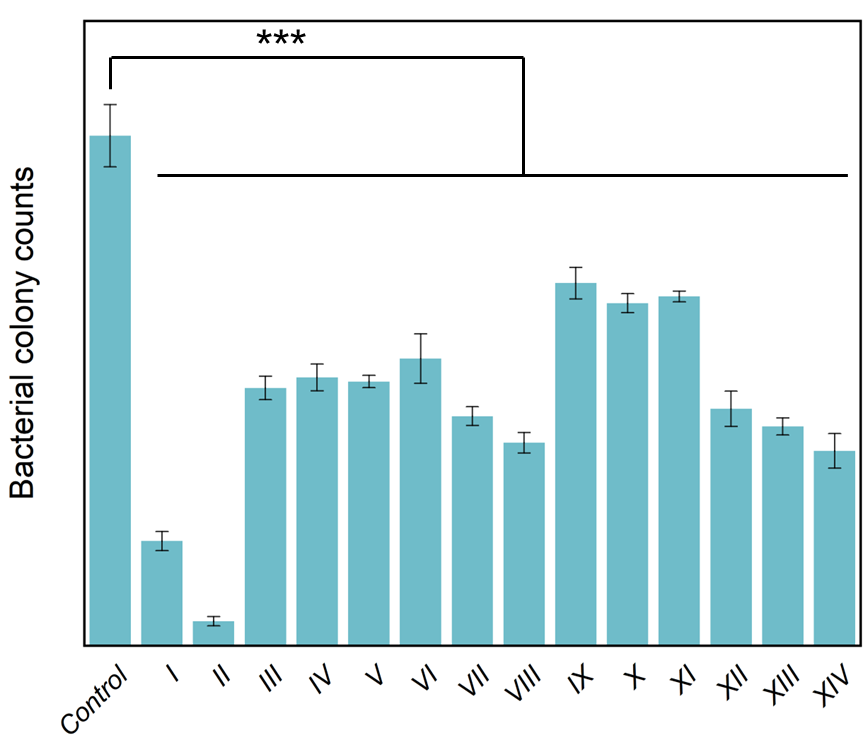


**Figure S7** Statistical analysis of bacterial colony counts on the D-MgCuPdGd alloy library. Data represent mean ± standard deviation. **p* < 0.05, ***p* < 0.01, and ****p* < 0.001.


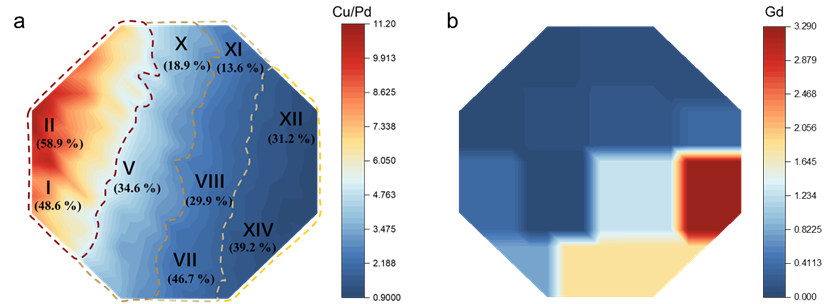


**Figure S8** (a) Antimicrobial efficiency map of MgCuPdGd alloy film without surface nanostructures, plotted as a function of the Cu/Pd atom ratio. (b) The content of Gd ion release from the MgCuPdGd alloy library film.


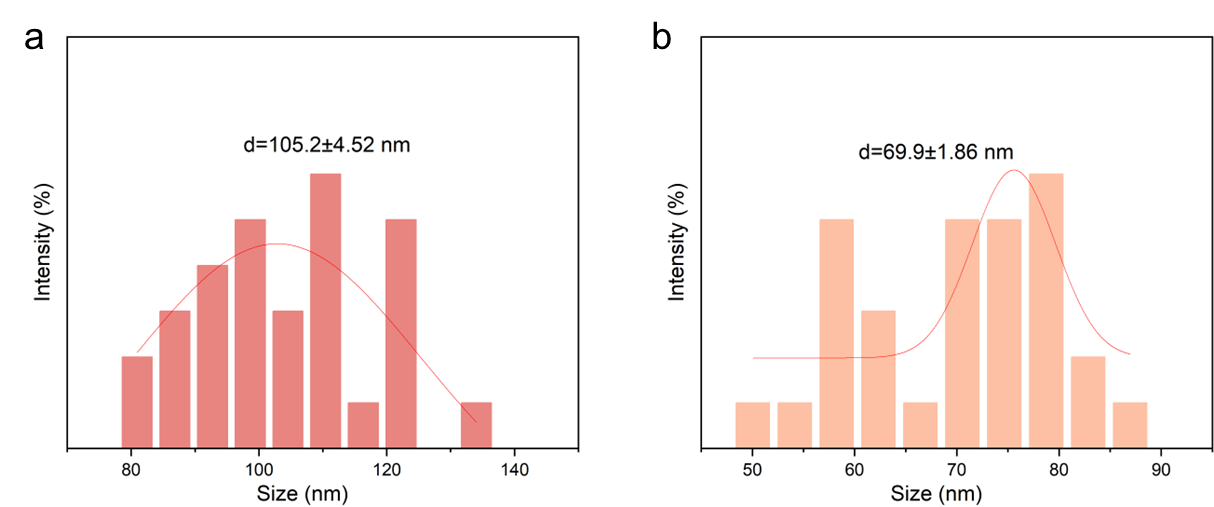


**Figure S9** Statistics of surface nanoparticles size in Zones I and II.


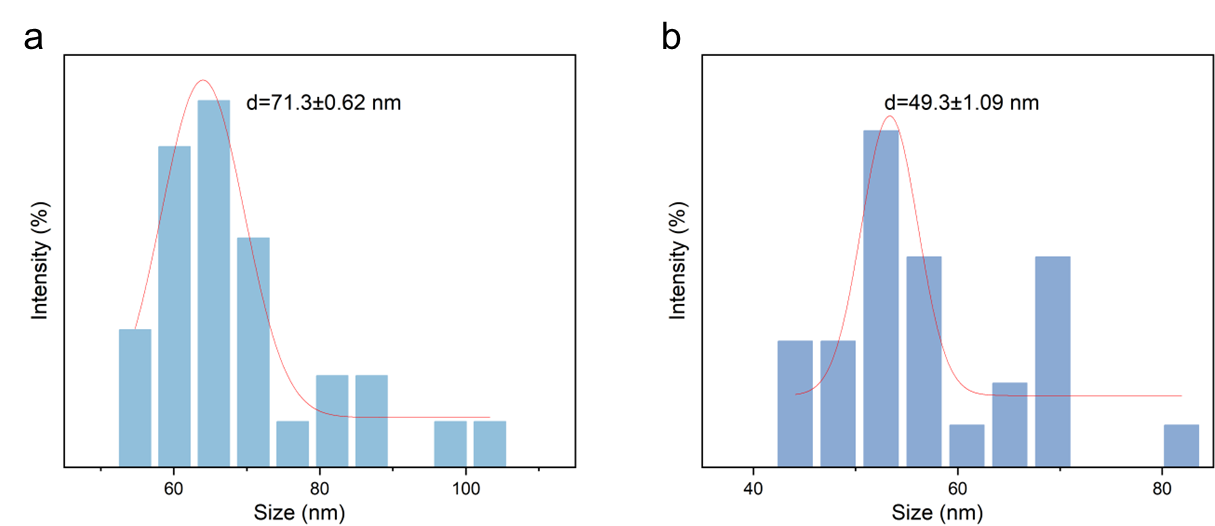


**Figure S10** Statistics of surface nanoparticles size in Zones V and X.


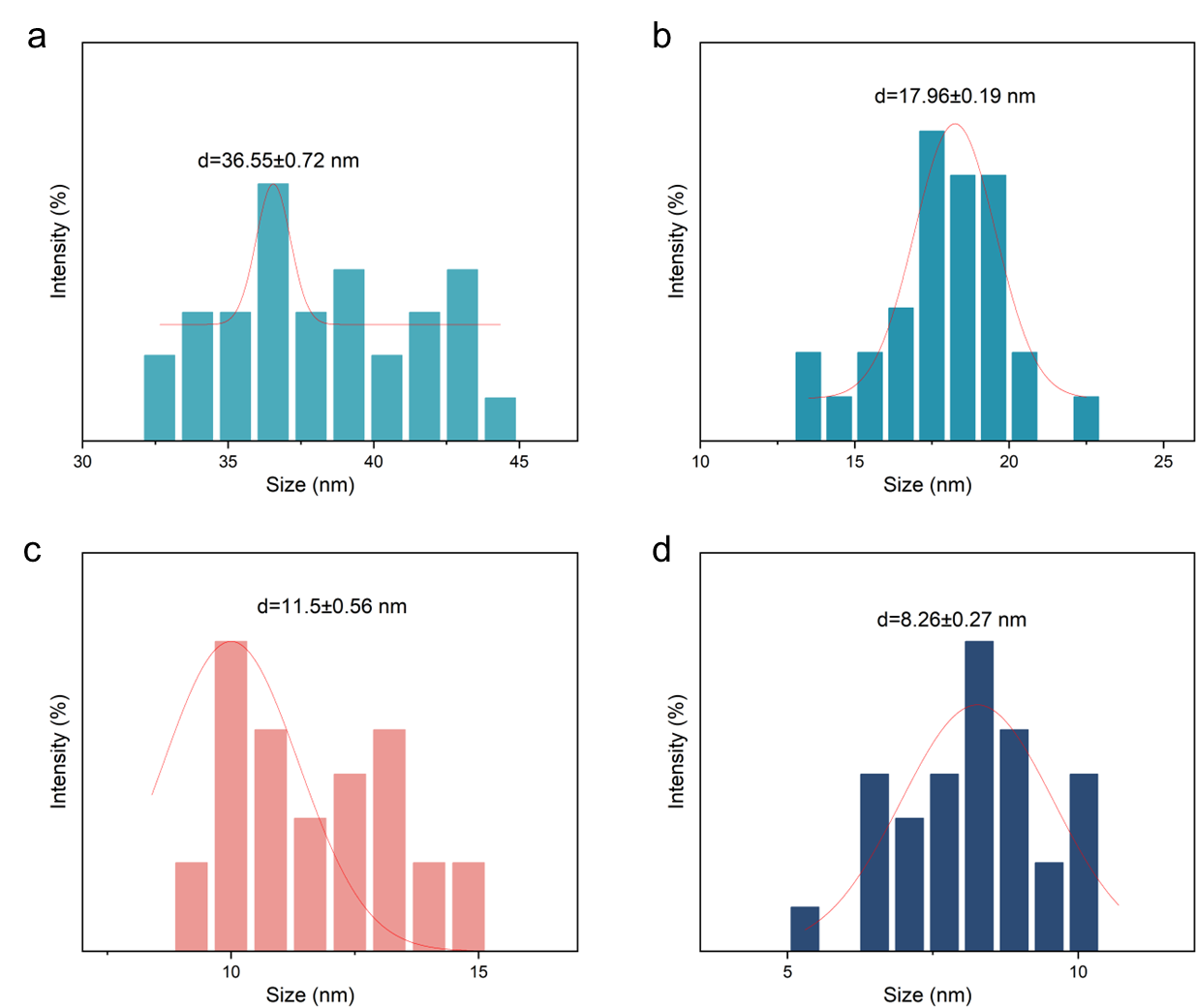


**Figure S11** Statistics of surface nanoparticles size in Zones XI, VIII, XII and XIV, respectively.


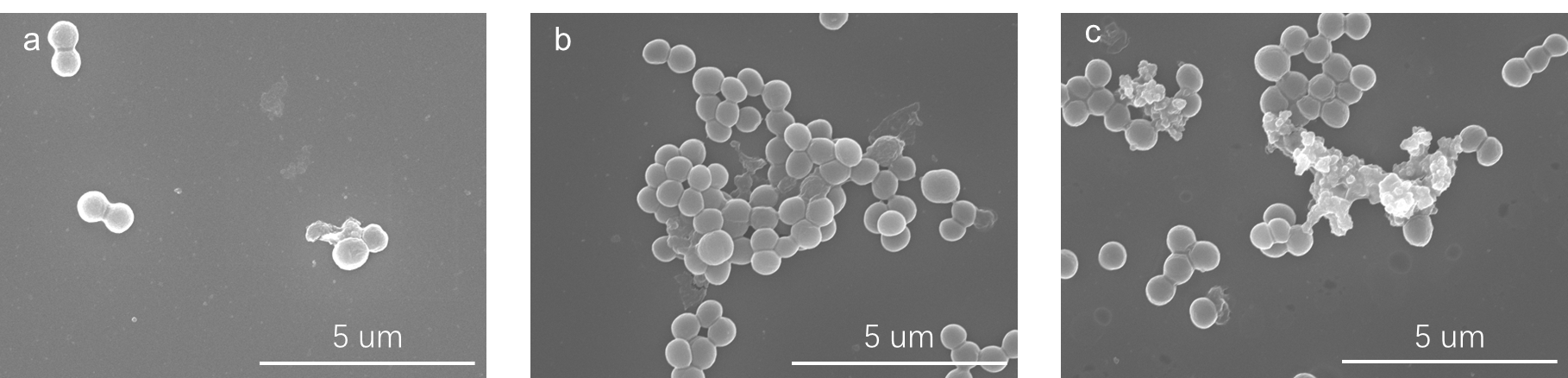


**Figure S12** SEM images of bacteria after contact with Zones II, X and XIV.


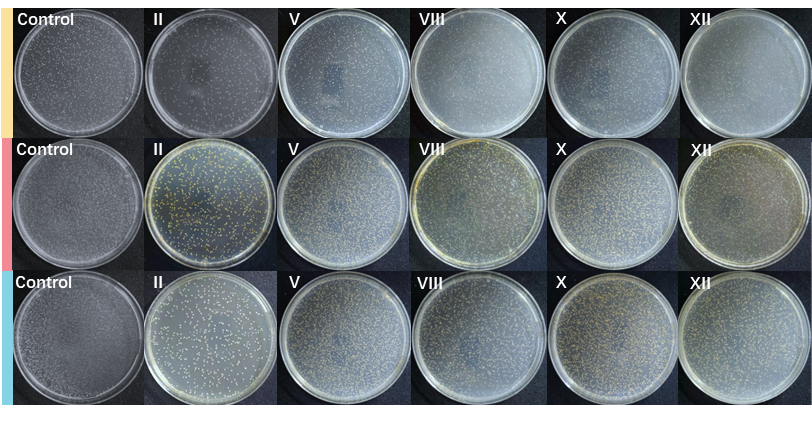


**Figure S13** Photographs of *S. aureus* bacteria colonies. Yellow, red and blue rows indicate colonies after 4, 8, and 12 h of co-culture with the films.


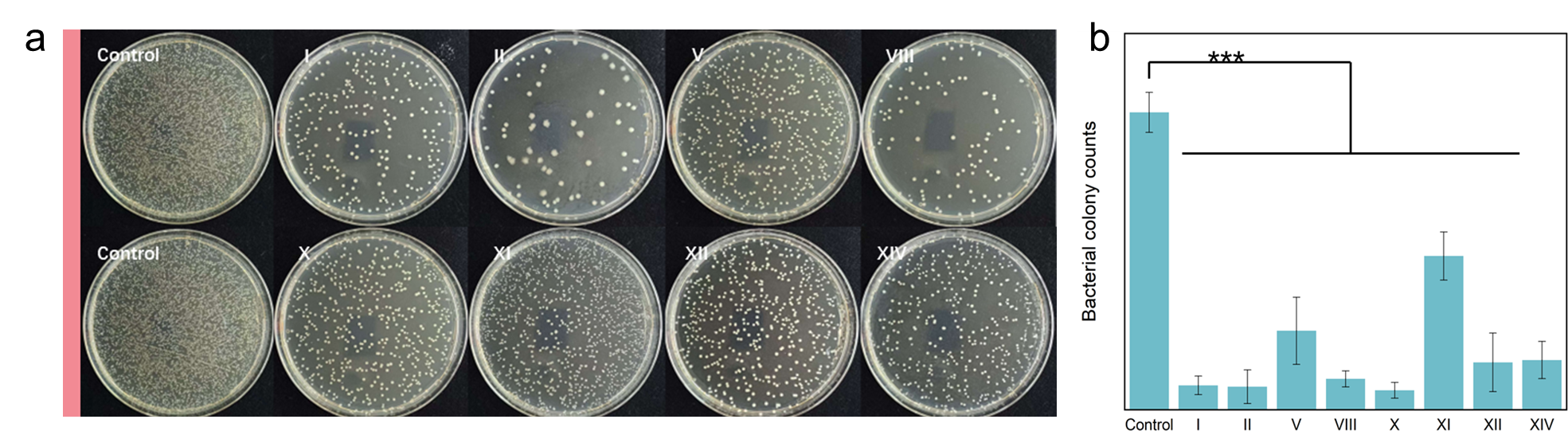


**Figure S14** (a) Photographs of *E. coli* colonies. (b) Statistical analysis of *E. coli* colony counts from the D-MgCuPdGd alloy library in Zones I, II, V, VIII, X, XI, XII, and XIV. **p* < 0.05, ***p*< 0.01, and ****p* < 0.001.


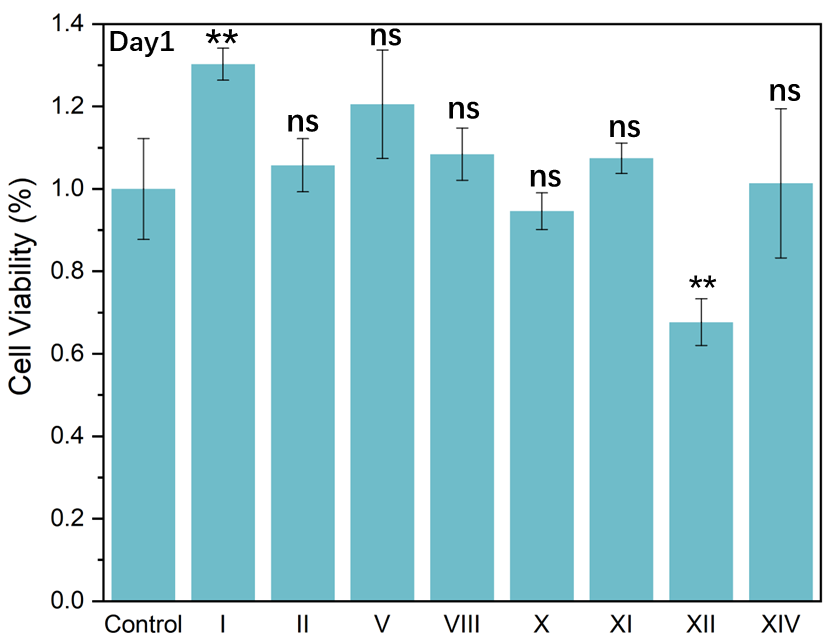


**Figure S15** Cytotoxicity of the D-MgCuPdGd alloy films in Zones I, II, V, VIII, X, XI, XII, and XIV after 1 day of incubation. **p* < 0.05, ***p*< 0.01, and ****p* < 0.001.

**Table S1** Ion release from D-MgCuPdGd film.

| Zone | Mg [μg mL^-1^] | Cu [μg mL^-1^] | Pd [μg mL^-1^] | Gd [μg mL^-1^] |
| --- | --- | --- | --- | --- |
| II | 14.3 | 1.51 | 0.141 | 0.7896 |
| V | 12.98 | 0.824 | 0.0014 | 0.0055 |
| VII | 6.93 | 0.4878 | 0.2678 | 13.32 |
| X | 10.89 | 0.5249 | 0.0304 | 0.3702 |
| XI | 4.47 | 0.0551 | 0.0623 | 0.3717 |
| XII | 2.16 | 0.52 | 0.1498 | 0.7127 |
| XIV | 2.73 | 0.9335 | 0.5048 | 19.32 |

**Table S2** Ion release from MgCuPdGd film.

| Zone | Mg [μg mL^-1^] | Cu [μg mL^-1^] | Pd [μg mL^-1^] | Gd [μg mL^-1^] |
| --- | --- | --- | --- | --- |
| II | 8.208 | 0.135 | 0.0011 | 0.00217 |
| V | 6.25 | 0.0255 | 0.0094 | 0.00483 |
| VII | 2.548 | 0.109 | 0.00998 | 1.8246 |
| X | 2.078 | 0.04 | 0.00116 | 0.07498 |
| XI | 2.066 | 0.0353 | 0.0089 | 0.0689 |
| XII | 0.8 | 0.08 | 0.05 | 0.3469 |
| XIV | 2.594 | 0.0744 | 0.09 | 3.289 |
